# Optimization of Ultrasound-Assisted Extraction of Morphine from Capsules of *Papaver somniferum* by Response Surface Methodology

**DOI:** 10.1155/2015/796349

**Published:** 2015-03-16

**Authors:** Ibrahim Bulduk, Bahdışen Gezer, Mustafa Cengiz

**Affiliations:** ^1^Uşak University, Department of Chemical Engineering, 64200 Uşak, Turkey; ^2^Süleyman Demirel University, Department of Chemistry, 32000 Isparta, Turkey

## Abstract

In this study, amount of morphine from poppy capsules *(Papaver somniferum)* was investigated using ultrasonic assisted extraction (UAE). Response surface methodology was used to estimate effective experimental conditions on the content extraction of poppy capsules. For this purpose, solvent/solid ratio (10–20 mL/500 mg sample), pH (1–13), time (30–60 min), and temperature (30–50°C) were chosen as experimental variables. The affected response is extraction recovery values for morphine from poppy straw. For interpreting the relationship between experimental factors and response, a design table was established with combinations of three different concentrations levels of this compound in 29 trials. The second order quadratic model gave a satisfactory description of the experimental data. In our study, *R*-Squared (0.96), Adj-*R*-Squared (0.92), and Pred *R*-Squared (0.78) values for extraction yield display good accuracy of the derived model. The predicted optimal conditions for the highest morphine level (3.38 mg morphine/500 mg-sample) were found at 19.99 mL solvent/500 mg solid ratio, 59.94 min extraction time, 1.10 pH, and 42.36°C temperature. In the optimal extraction conditions, the experimental values are very close to the predicted values. Consequently, the response surface modeling can be achieved sufficiently to predict extraction yield from poppy straw by ultrasound assisted extraction.

## 1. Introduction

**Figure 1:**
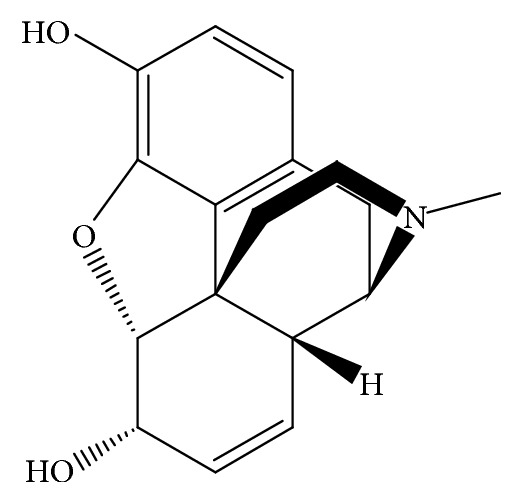
The structure of morphine.

*Papaver somniferum L*. is known as opium poppy, which has been used as a medicinal remedy for its sedative, anesthetic, analgesic, and antidierrheal effects for thousands of years. There are ancient documents mentioning about the medicinal properties of opium poppy such as the “De Materia Medica” written in A.C. 65 by Pedanius Dioscorides [1]. By the more recent studies, it is known that the medicinal properties of opium poppy come from its high concentration alkaloid content. Morphine, codeine, thebaine, papaverine, and noscapine are important and effective alkaloids in opium poppy for medicinal usage. Besides their medicinal properties, these alkaloids are highly addictive and thus they have forensic importance [2]. For instance, morphine is a raw ingredient of illegal heroine. Morphine is a benzylisoquinoline alkaloid with two additional ring closures.  Figure 1 shows molecular structure of morphine. Therefore, appropriate analysis of alkaloid content of opium poppy for screening and effective extraction is of great importance. Furthermore, the alkaloid contents present in opium poppy make it an asset for the researchers. So we hope these findings will be helpful and directive for us and/or other scientists in the future research of* Papaver* species.

Conventional extraction methods require more solvent, higher temperatures, and longer periods when high efficiency is desired [3]. Nowadays, extraction techniques which can be applied in lower temperatures requiring less amount of solvent and time are looking for by the researcher. The ultrasonic extraction technique (UAE), using ultrasound waves disrupt open the cellular walls, accomplishes mass transfer successfully [4, 5]. By efficiently accomplishing mass transfer, ultrasound assisted extraction proves itself to be an efficient way for extraction. When compared to another extraction techniques such as microwave and supercritical extraction UAE is cheaper and simpler [5, 6]. Recently, UAE has become a popular technique among most researchers who try to extract flavonoids, polysaccharides, polyphenols, and oil from different matrices [5, 7]. However, not only the extraction method but also the other parameters such as preliminary preparations, particle size of the extracted material, solvent type, solvent concentration, and solvent/solid ratio, extraction temperature, extraction pressure, extraction time, and pH affect the extraction process. To obtain the optimal operating conditions for an extraction method is necessary for commercial applications of the process. In the literature, there are two optimization method in order to optimize the process. One of them is one-factor-at-a-time approach; and the other one is response surface methodology. One factor at a time is classical method, time consuming, and expensive method. Moreover, by ignoring possible interactions among other operating parameters, this approach may result in misleading conclusions. In fact, the response of a process occurs by the interactions of different variables which affect the operation. Response surface methodology (RSM) considers the probable interactions among operating parameters. In order to simplify this process and maximize utilization of data, design of experiments (DoE) approach can be used and then the response surface methodology (RSM) can be applied for the experimental results [5, 8]. Utilization of DoE and RSM enables us to reduce the number of experiments needed and accurately estimate the optimal conditions from the experimental data [5].

About the poppy straw: the various reported morphine extraction methods from poppy straw have been reported in the literature. These reported methods include various steps involving water, organic solvent, and pH adjustments. The simplest methods are the following ones covering the sonication: even these simplest methods contain some more purification and organic solvent stage. Individual alkaloids (morphine, codeine, papaverine, noscapine, thebaine, oripavine, reticuline, and narceine) were quantitatively determined in the different samples by a validated capillary electrophoresis method [9].

Depending on the plant structure, cultivar, and harvesting time, the composition of morphine is changeable. Therefore, a common extraction procedure for all plant species cannot exist and for poppy straw. It should be designed and optimized. Particle size of the extracted material, solvent type, solvent composition, the solvent/solid ratio, extraction temperature, extraction pressure, extraction time, and pH of the solvent are the parameters affecting the industrial processes which are applied for extraction. In order to optimize the process, possible interactions among other operating parameters should be considered. Response surface methodology (RSM) considers the probable interactions among operation parameters. RSM is an aggregate of statistical and mathematical techniques used for developing, improving, and optimizing the processes [5, 10]. Sonication has been applied for the extraction of polycyclic aromatic hydrocarbons, pharmaceutically active compounds, and flavonoids from different matrices [5, 11, 12].

The aim of this study is to determine the effect of applied parameters on poppy straw by using ultrasound extraction method with RSM.

## 2. Experimental

### 2.1. Reagents and Materials

*Papaver somniferum* was collected from fields of Opium Alkaloids Factory on the harvesting period in July of 2012 (Turkey). Capsules of the plant were dried at room temperature after the seeds within the plant had been removed. Dried capsules were grinded to the size of 80–100 mesh before extraction.

All chemicals used in experiments were of analytical grade and all chemicals used for analysis were of HPLC grade. Analytical grades NaOH and HCl were purchased from Merck Chemicals. 0.45 *μ*m membranes (Millipore, Bedford, MA, USA) was used for filtering the buffer solutions. Morphine Sulfate USP Reference Standard CII (Cat. number 1448005) was obtained from the Turkish Grain Board (Afyonkarahisar, Turkey), which is the only legal supplier of narcotics in Turkey.

Specifications of Morphine Sulfate USP Reference Standard CII (Cat. number 1448005):

 Molecular formula of morphine sulfate is (C_17_H_19_NO_3_)_2_·H_2_SO_4_·5H_2_O.
 Molecular weight of morphine sulfate is 758.83.
 132.93 mg of morphine sulfate pentahydrate is equivalent to 100 mg of anhydrous morphine base.

### 2.2. Ultrasound Assisted Extraction

Ultrasound assistant extraction was carried out using Bandelin Sonorex brand ultrasonic bath with 50 kHz frequency. pH levels of the solutions were controlled by Mettler Toledo—Seven Easy pH meter. For the standard ultrasonic conditions, Erlenmeyer flasks were placed inside the ultrasonic bath. Water inside the ultrasonic bath was circulated in order to keep the temperature stable. Solvent level in the Erlenmeyer flask and water level in the ultrasonic bath were kept the same. In order to adjust pH levels to 1 or 13, hydrochloric acid and sodium hydroxide were used. After the extraction process had been completed, mixture was filtered with Whatman filter paper in order to prevent capillary blockage first and was then filtered with 0.45 micron membrane filter (Millipore, Bedford, MA, USA).

### 2.3. Chromatographic Conditions (HPLC Method) [13]

Identification and quantitative determination of morphine was established by Agilent 1260 chromatographic system equipped with autosampler, quaternary pump, column compartment, and a multiwavelength detector. Final quantification was performed on a 250 mm × 4.6 mm id, 5 *μ*m particle size, Zorbax Extend C-18 column (Agilent Technologies). The eluents were a solution of 0.1% TFA in water and pH adjusted to 9.6 with TEA (solvent A) and methanol (solvent B), both filtered through 0.45 *μ*m Millipore filters. Elution was performed with the gradient: 0–2 min 45% solvent B; 2–10 min from 45 to 70% solvent B; 10–20 min from 70 to 85% solvent B; 20–20.1 min from 85 to 45% solvent B; 20.1–30 min 45% solvent B. The flow rate was 1.5 mL/min and the injection volume was 20 *μ*L. The column temperature was maintained at 30°C, and detection was carried out at 280 nm [13].

The method has been validated according to ICH guidelines, taking into account the recommendations of other appropriate guidelines. Results obtained from testing different parameters during validation of the analytical method were given in Table 1. The method recommended by the European Pharmacopoeia for the quality assessment of the opium dry extract has been standardized (Ph. Eur. 7.0: 01/2008 : 1839). This method analyzes not only morphine but also other poppy alkaloids [13].

The analysis method is in gradient conditions. In addition to morphine, codeine, oripavine, thebaine, papaverine, and noscapine were analyzed by this method. Despite the retention time of last noscapine peak is 13. 5 minutes. Run time is 30 minutes. This is because the organic phase content of mobile phase increases with the analysis progresses. Organic content of mobile phase is the maximum at 20 minute. The column is cleaned by this process. Then, concentration of mobile phase will return to the initial conditions after 20 minutes. The analysis results of only morphine were given and optimized.

Calibration curve was created to determine the concentration of morphine by comparing the peak areas with standard solutions which consist of morphine in the range of 100–500 *μ*g/mL.

Morphine standard solution was prepared by dissolving its salt in purified water. It was injected as such. The samples were injected as stated in the experimental conditions. Morphine, codeine, thebaine, and noscapine were analyzed and optimized in poppy capsules; however optimization of other alkaloids were not included in this paper.

### 2.4. Experimental Design

Design-Expert suggestion Box-Behnken designs for three to seven factors. These designs require only three levels, coded as −1, 0, and +1. Box and Behnken created this design by combining two-level factorial designs with incomplete block designs. This procedure creates designs with desirable statistical properties, but, most importantly, with only a fraction of the experiments needed for a full three-level factorial. These designs offer limited blocking options, except for the three-factor version. In this design, while two of the factors are constant at level 0, the remaining factors are iterated within the levels +1 and −1; then this process is repeated for different groups [14, 15]. In this study, Box-Behnken design was performed with four parameters to explore the effect of them on the responses (Table 2). These were *X*1 (solvent/solid ratio, mL/500 mg), *X*2 (pH), *X*3 (time, min), and *X*4 (temperature, °C). Moreover, the response was extraction yield (EY). Design-Expert 8.0.7.1 (trial version) software was used in order to apply the Box-Behnken design model. Twenty-nine experiments were performed with three replications at the center values to evaluate the pure error sum of squares.

Experimental data were fitted to the quadratic model. The proposed quadratic model is shown as follows:

(1) $$\begin{array}{c} Y=\beta_{0}+\overset{4}{\underset{i=1}{\sum }}\beta_{i}X_{i}+\overset{4}{\underset{i=1}{\sum }}\beta_{ii}X_{i}^{2}+\overset{4}{\underset{i=1}{\sum }}\overset{4}{\underset{j=i+1}{\sum }}\beta_{ij}X_{i}X_{j}+e, \end{array}$$

where *Y* is the response, *β*_0_ is the constant coefficient which is often described as intercept, *X*_*i*_ (*i* = 1 − 4) is the noncoded variable, *β*_*i*_ is the linear, and *β*_*ii*_ is the quadratic, and (*i* and *j* = 4) *β*_*ij*_ are the second order interaction coefficients [5, 16].

### 2.5. Statistical Analysis

Statistical analysis on the means of triplicate experiments was performed out using the variance analysis (ANOVA) procedure of the Instat software version 3.0. Variance analysis were applied to identify the interaction between the variables and the response using Design-Expert program. Three replication analyses were carried out for each sample. Variance analysis were applied for identifying the interaction between the variables and the response by using Design-Expert program. The results of HPLC analysis were expressed as means of extraction yield.

## 3. Results and Discussions

### 3.1. Effect of Process Variables on the UAE Performance

**Figure 2:**
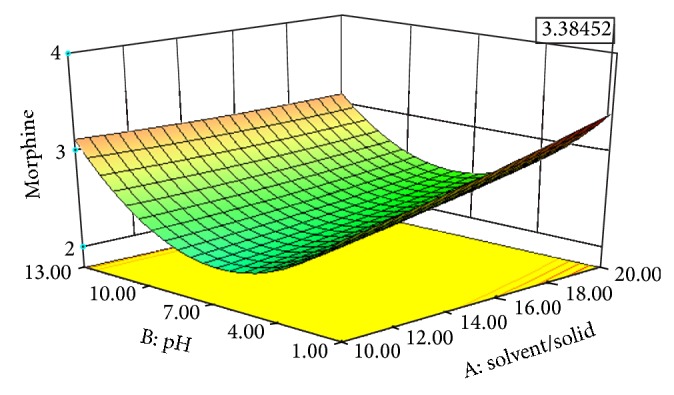
Response surface plot for the EY of poppy straw as a function of solvent/solid ratio (*X*1) to solvent pH (*X*2).

**Figure 3:**
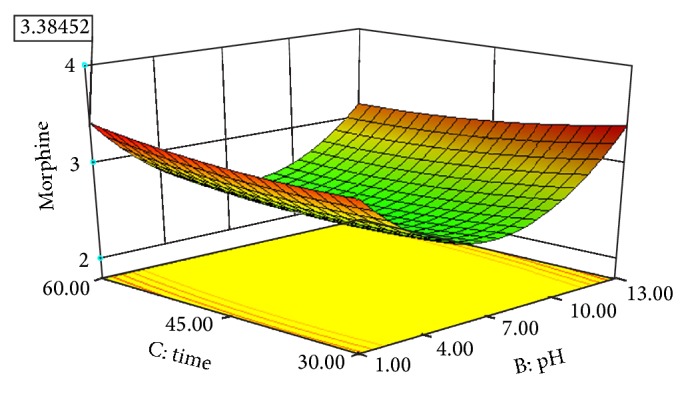
Response surface plot for the EY of poppy straw as a function of solvent pH (*X*2) to extraction time (*X*3).

**Figure 4:**
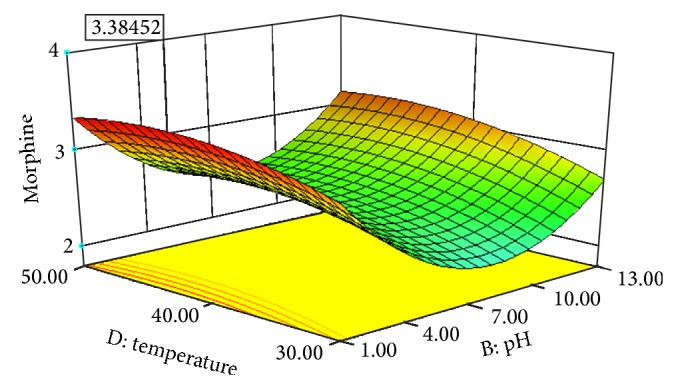
Response surface plot for the EY of poppy straw as a function of solvent pH (*X*2) to extraction temperature (*X*4).

**Figure 5:**
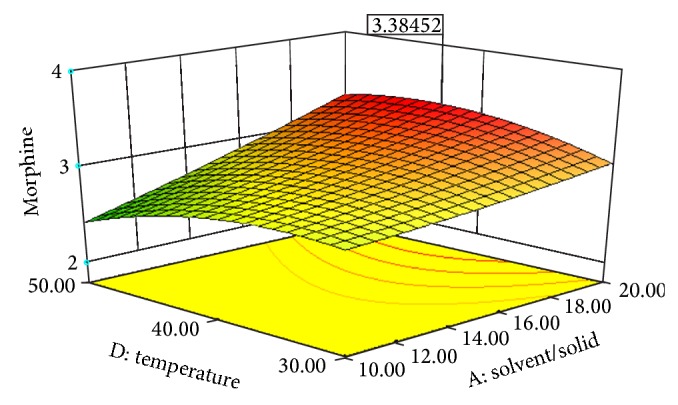
Response surface plot for the EY of poppy straw as a function of solvent/solid ratio (*X*1) to extraction temperature (*X*4).

**Figure 6:**
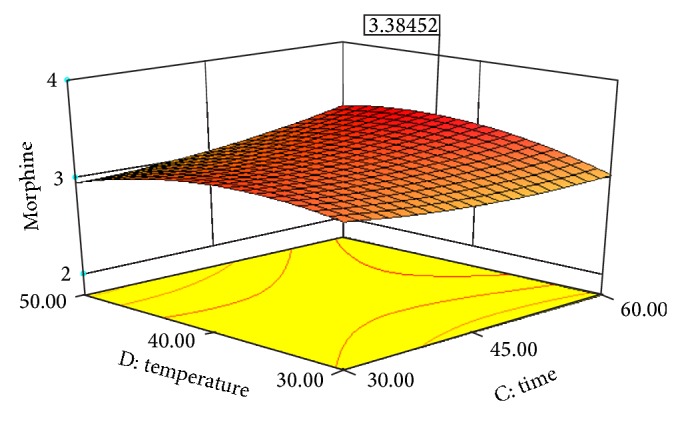
Response surface plot for the EY of poppy straw as a function of extraction time (*X*3) to extraction temperature (*X*4).

**Figure 7:**
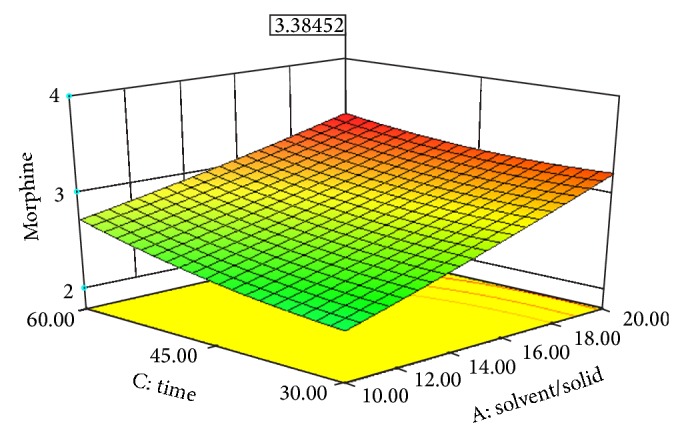
Response surface plot for the EY of poppy straw as a function of solvent/solid ratio (*X*1) to extraction time (*X*3).

Experimental conditions of Box-Behnken design runs designed with Design Expert 8.0.7.1 are shown in Table 3. Table 3 also displays the effects of pH, solvent/solid ratio, time and temperature on the extraction efficiency obtained by UAE. Figures 2, 3, 4, 5, 6, and 7 display response surface plots for process variables.

### 3.2. Effect of Solvent/Solid Ratio on the UAE Performance

The solvent/solid ratio is a crucial factor and was also studied to optimize extraction efficiency. Large solvent volumes could make the procedure difficult and lead to unnecessary waste, while small volumes may lead to incomplete extraction. A series of experiments were carried out with different solvent/solid ratios (10/500, 15/500, 20/500 v/w) to evaluate the effect of the solvent/solid ratio. Table 3 shows that the extraction efficiency increased with increasing solvent volume. The increase of the yield with the increase of solvent quantity is consistent with mass transfer principles, since the concentration gradient which is driving force is supposed to be higher when a lower solvent to solid ratio is used, leading to higher diffusion.

### 3.3. Effect of Solvent pH on the UAE Performance

The influence of the solvent pH on the extraction efficiency of morphine was examined over a range of 1–13 and the results are shown in Table 3. It can be clearly seen that extraction efficiency was the maximum at pH: 1. When the solvent pH was increased from 1 to 7, the extraction efficiency decreased and was the minimum at pH: 7. Then the extraction efficiency increased gradually, while the solvent pH was increased from 7 to 13. This might be explained by the fact that an acidic environment helps in the release of morphine into the solvent.

### 3.4. Effect of Extraction Time on the UAE Performance

The influence of the time ultrasound on the extraction efficiency of morphine was examined over a range of 30–60 min and the results are shown in Table 3. They show that when the extraction time was increased from 30 min to 60 min, the extraction efficiency was low during the first 40 min of ultrasonication indicating that more time was needed for ultrasound to disrupt the cell walls and aid the release of morphine into the solvent.

### 3.5. Effect of Extraction Temperature on the UAE Performance

Extractions of poppy straw were performed over temperatures ranging from 30 to 50°C, since extraction at high temperatures is not economical. Expectedly, extraction efficiency of morphine increased steadily as a function of temperature until 45°C, since mass transfer increase by temperature. But extraction efficiency of morphine decreased slowly after 45°C. In general, extractions at higher temperatures increase mass transfer and extraction performance because of enhanced solute desorption from the active sites of plant matrix.

### 3.6. Optimisation of UAE by RSM

Individual effects of process variables, which are also known as one-factor-at-at-ime approach, were applied in previous section. This classical approach ignores the possible interactions of process variables with each other, which may result in misleading conclusions. Response surface methodology (RSM) considers the probable interactions between operation parameters. Table 2 shows the four parameters (pH, solvent/solid ratio, time, and temperature) including minimum, centre, and maximum points. Twenty-nine experiments were run and chosen randomly by the design expert software, and the responses were recorded (Table 3). Using response surface methodology owing to the software, a quadratic model applying not only forward stepwise but also backward elimination regressions for EY was obtained.

Using responce surface methodology from the software, a quadratic model given below was derived:

(2) M=3.000280−0.040567X1−0.172330X2 −0.035744X3+0.038358X4−0.005500X1X2 −0.000933X1X3+0.003200X1X4 −0.001167X2X3+0.000708X2X4 +0.000783X3X4+0.00104X12+0.021387X22 +0.000311X32−0.001713X42.

In Table 4, *X*3, *X*1*X*3, *X*2*X*3, *X*2*X*4, *X*3*X*4, *X*1^2^, and *X*3^2^ are not significant effects for the model. After excluding their regression coefficients, new model may be given for better explanation of new condition:

(3) M=3.000280−0.040567X1−0.172330X2 +0.038358X4−0.005500X1X2+0.003200X1X4 +0.021387X22−0.001713X42.

**Figure 8:**
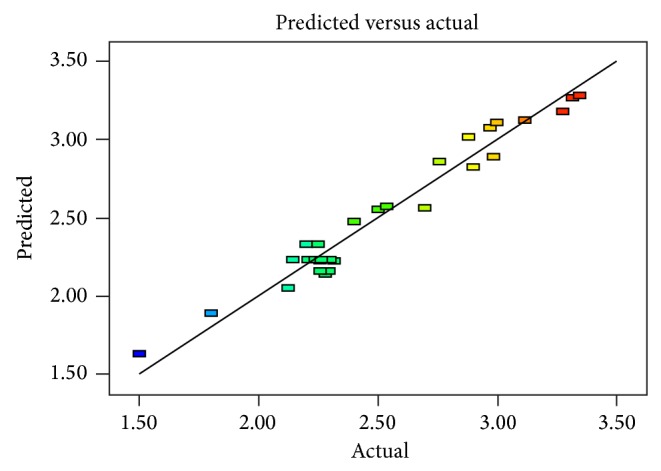
The correlation between the experimentally obtained values of the EY versus the calculated values using the model equation.

Theoretical recovery values for morphine calculated from this equation were plotted against practical ones. These relationships were shown in Figure 8.

The optimal extraction conditions were found by using optimization choice in design expert software to maximize the response. This value was measured at 1.10 of pH, 60 min of extraction time, 42.36°C of extraction temperature, and 19.99 mL to 500 mg solvent/solid ratio. The maximum response was found as 3.38 mg morphine/500 mg dried capsules under these operating conditions.

After finding optimal conditions, real sample extraction experiments were repeated 6 times and then average with relative standard deviation was calculated.

 Average is 3.362.
 Standard deviation is 0.015.
 Relative standard deviation is 0.446.
 Morphine yield (mg/500 mg poppy capsules) is 3.362 ± 0.015.
 Results are appropriate for the statistical evaluation.

### 3.7. Model Fitting

The results of variance analysis were given in Table 4. In order to obtain the most suitable set of variables, stepwise regression was used. According to this regression, variables are tested and evaluated within the given alpha levels (0.1) using both backward and forward techniques. Backward techniques cover all the variables to estimate parameters and then any variables with a nonsignificant parameter at alpha levels are removed from the equation. This process continues until there are no significant variables left. Similar to backward technique, forward technique also evaluates the given variables within the given alpha levels. Unlike backward technique, forward technique starts with no variables included in the equation. The significant variable with the highest value of standardized beta (*P* < 0.05) will be added to the equation. Then the next variable with the highest standardized beta value is assessed. If the variable is significant, it is added to the equation. This process continues until no significant variables are left. Two of these regressions gave the same results [5, 17].

Variance analysis for the quadratic equations of Design Expert 8.0.7.1 for the response were given in Table 4. Regression analysis were performed at 95% of confidence interval. *F*-value of the derived model is 23.46 and *P* < 0.0001 indicates that derived model is significant. (*X*1), (*X*2), (*X*4), (*X*1*X*2), (*X*1*X*4), (*X*2^2^), and (*X*4^2^) are significant model terms in the confidence interval (Table 4). The closer and higher multiple coefficients (*R*-Squared, Adj *R*-Squared, and Pred *R*-Squared) point out to the higher accuracy of the model. Adj *R*-Squared also shows a high degree of correlation between actual and predicted data. As seen in Table 4 solvent/solid ratio (*X*1) is the most significant variable on the response. The “*F*-value” of “lack of fit” (6.15) shows that the lack of fit is significant.

In our study, *R*-Squared (0.96), Adj *R*-Squared (0.92), and Pred *R*-Squared (0.78) values for EY display good accuracy of the derived model. Also, the coefficient value of variation (C.V. %) was found as 5.20, respectively. The lower coefficient of variation value indicates a higher precision and reliability of the experimental results [18].

**Figure 9:**
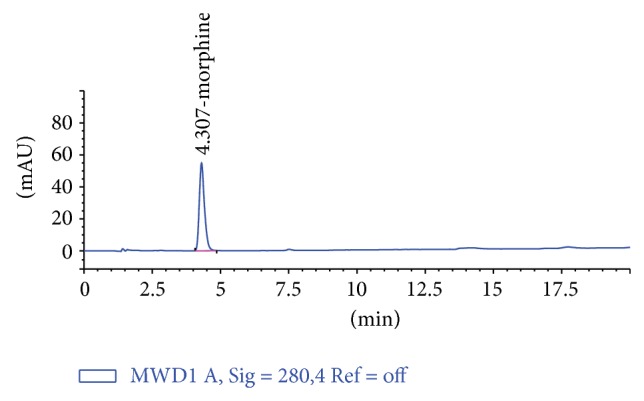
Chromatogram of the morphine standard solution (concentration: 200 *μ*g/L).

**Figure 10:**
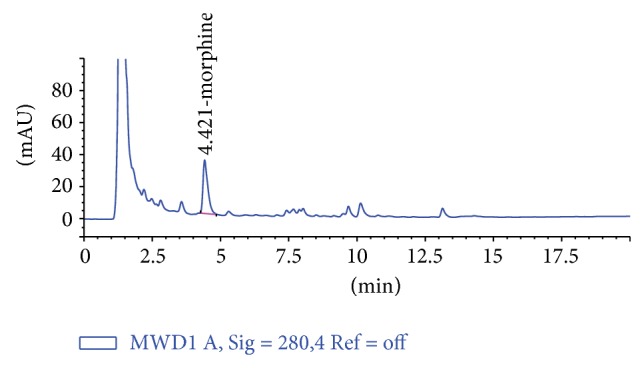
Chromatogram of poppy straw extract (run: 4, concentration: 164 *μ*g/L).

Hereby, the responce surface modeling can be accomplished sufficiently to predict EY from poppy straw with UAE. The lower value of coefficient of variation indicates a higher precision and reliability of the experimental results [19, 20]. The coefficient value was found to be 5.20 in our study. Figure 8 exhibits the corelation between the experimental and predicted data calculated from (3) concerning the EY of poppy straw extracts obtained by UAE. It can be seen that the predicted date calculated from the model is in good agreement with the experimental data in the range of operating conditions.  Figure 9 exhibits chromatograme of morphine standard solution.  Figure 10 exhibits chromatograme of poppy straw extract.

## 4. Conclusions

A potential source of morphine, poppy straw (*Papaver somniferum capsules*), was used as the research material in this study. The capsules were extracted by UAE, which is an environmental and economical alternative to conventional extraction methods, requiring more time and solvent consumption. The results of the study suggests that 1.10 of pH, 42.36°C of extraction temperature, 59.94 min of extraction time, and 19.99 mL to 500 mg solvent/solid ratio should be employed as optimal operating conditions for the best EY (3.38 mg morphine/500 mg dried capsules). Linear coefficient of solvent to solid ratio, pH and extraction time, and square coefficient of solvent to solid ratio, pH, and extraction time have the most significant effect on the EY obtained by UAE. After finding optimal conditions, real sample extraction experiments were repeated 6 times and then average with relative standard deviation was calculated. Morphine yield (mg/500 mg poppy capsules) is 3.362 ± 0.015. Results are appropriate for the statistical evaluation.

## Figures and Tables

**Table 1:** Results obtained from testing different parameters during validation of the analytical method.

Parameters	Morphine
Specificity	
Peak purity ratio	0.001
Linearity	
Concentration range *μ*g/mL	50–500
Correlation coefficient	0.9993
Intercept	−16.598
Slope	3.711
LOD	1.8
LOQ	5.4
Retention time	4.4

**Table 2:** Values of the independent variables and their coded forms with their symbols employed in RSM for optimization of poppy straw through UAE.

Independent variables	Units	Symbols of the variables	Coded levels		
			−1	0	1
Solvent/solid ratio	mL/500 mg	(*X*1)	10	15	20
pH	pH	(*X*2)	1	7	13
Extraction time	min	(*X*3)	30	45	60
Extraction temp.	°C	(*X*4)	30	40	50

**Table 3:** Box-Behnken design of the independent variables (*X*1, *X*2, *X*3, *X*4) and experimental results for the EY.

Number	*X*1 solvent/solid ratio	*X*2 pH	*X*3 time	*X*4 temperature	EY of morphine^*^
	mL/500 mg	pH	min	°C	mg/500 mg poppy straw
1	10 mL/500 mg	1	45	40	2.40
2	20 mL/500 mg	1	45	40	3.32
3	10 mL/500 mg	13	45	40	3.12
4	20 mL/500 mg	13	45	40	3.28
5	15 mL/500 mg	7	30	30	2.25
6	15 mL/500 mg	7	60	30	2.28
7	15 mL/500 mg	7	30	50	1.80
8	15 mL/500 mg	7	60	50	2.30
9	10 mL/500 mg	7	45	30	2.26
10	20 mL/500 mg	7	45	30	2.32
11	10 mL/500 mg	7	45	50	1.50
12	20 mL/500 mg	7	45	50	2.12
13	15 mL/500 mg	1	30	40	2.90
14	15 mL/500 mg	13	30	40	3.35
15	15 mL/500 mg	1	60	40	2.97
16	15 mL/500 mg	13	60	40	3.00
17	10 mL/500 mg	7	30	40	2.12
18	20 mL/500 mg	7	30	40	2.54
19	10 mL/500 mg	7	60	40	2.26
20	20 mL/500 mg	7	60	40	2.40
21	15 mL/500 mg	1	45	30	2.76
22	15 mL/500 mg	13	45	30	2.88
23	15 mL/500 mg	1	45	50	2.81
24	15 mL/500 mg	13	45	50	2.99
25	15 mL/500 mg	7	45	40	2.24
26	15 mL/500 mg	7	45	40	2.31
27	15 mL/500 mg	7	45	40	2.07
28	15 mL/500 mg	7	45	40	2.21
29	15 mL/500 mg	7	45	40	2.27

^*^Data are expressed as the mean (*n* = 3).

**Table 4:** The analysis of variance (ANOVA) for the quadratic equations of design expert 8.0.7.1 for the EY.

Source	Sum of squares	df	Mean square	*F*-value	*P* value prob. > *f*
Model	5.65	14	0.40	23.46	<0.0001
*X*1-Slv./Sld.	0.44	1	0.44	25.62	0.0002
*X*2-pH	0.18	1	0.18	10.46	0.0060
*X*3-Time	0.0052	1	0.0052	0.30	0.5909
*X*4-Temp.	0.13	1	0.13	7.69	0.0150
*X*1*X*2	0.11	1	0.11	6.33	0.0247
*X*1*X*3	0.020	1	0.020	1.14	0.3039
*X*1*X*4	0.10	1	0.10	5.95	0.0286
*X*2*X*3	0.044	1	0.044	2.56	0.1317
*X*2*X*4	0.0072	1	0.0072	0.42	0.5275
*X*3*X*4	0.055	1	0.055	3.21	0.0949
*X*1^2^	0.0044	1	0.0044	0.26	0.6193
*X*2^2^	3.85	1	3.85	223.44	<0.0001
*X*3^2^	0.032	1	0.032	1.84	0.1961
*X*4^2^	0.19	1	0.19	11.07	0.0050
Residual	0.24	14	0.017	—	—
Lack of fit	0.23	10	0.023	6.15	0.0475
Pure error	0.015	4	0.0037	—	—
Cor. total	5.89	28	—	—	—

df, degrees of freedom.

Cor., totals of all information corrected for the mean.
